# Sulcal-Gyral Pattern in the Scalp Fibrous Hamartoma of Infancy: A Case Report

**DOI:** 10.7759/cureus.92907

**Published:** 2025-09-22

**Authors:** Talha Sajid, Faheem A Usmani, Sana Imdad, Shahrukh Rizvi, Abdul Majid

**Affiliations:** 1 Neurological Surgery, Punjab Institute of Neurosciences, Lahore, PAK; 2 Plastic Surgery, Lahore General Hospital, Lahore, PAK

**Keywords:** fibrous hamartoma of infancy, scalp mass, sulcal-gyral pattern, surgical excision, triphasic histology

## Abstract

Fibrous hamartoma of infancy (FHI) is a benign soft-tissue tumor that typically presents within the first two years of life. We report an eight-year-old boy with a long-standing, painless, left fronto-parietal scalp mass that enlarged over two years. Physical examination showed a 6×5×2 cm, firm, mobile, non-tender subcutaneous lesion with overlying hair. Magnetic resonance imaging (MRI) and contrast-enhanced computed tomography (CT) showed a subcutaneous, fat-containing mass without bony involvement and a sulcal-gyral surface pattern. The lesion, including overlying skin and thickened pericranium, was excised en bloc, and the defect closed with a rotational flap. Histopathology confirmed FHI, showing the characteristic triphasic composition of mature adipose tissue, fibrocollagenous fascicles, and primitive spindle-to-stellate cells in a myxoid stroma without atypia or necrosis. This case illustrates that FHI, although usually infantile and truncal, can occur on the scalp in older children, and that complete surgical excision is an effective management option.

## Introduction

Fibrous hamartoma of infancy (FHI) is a rare, benign soft-tissue lesion of early childhood that typically presents within the first two years of life and favors the trunk and proximal extremities [1,2]. Head-and-neck involvement is uncommon, and scalp cases are particularly rare [2,3]. Among the different locations of FHI, only 7.5% are found in the craniocervical region [4]. Radiologic imaging plays a critical role in diagnosing and characterizing FHI. Magnetic resonance imaging (MRI) often reveals distinct features such as heterogeneous low-signal masses, fatty components, and involvement of subcutaneous tissues [5]. Histopathologic confirmation rests on the characteristic triphasic pattern of mature adipose tissue, fibrocollagenous bundles, and primitive mesenchymal cells [2,6]. The most definitive and effective way of treatment is surgical excision of the lesion [5].

We report an eight-year-old with scalp FHI showing an unusual sulcal-gyral surface pattern that mimics cerebral convolutions, potentially complicating the assessment, yet aiding recognition when correlated with imaging and histopathology.

## Case presentation

**Figure 1:**
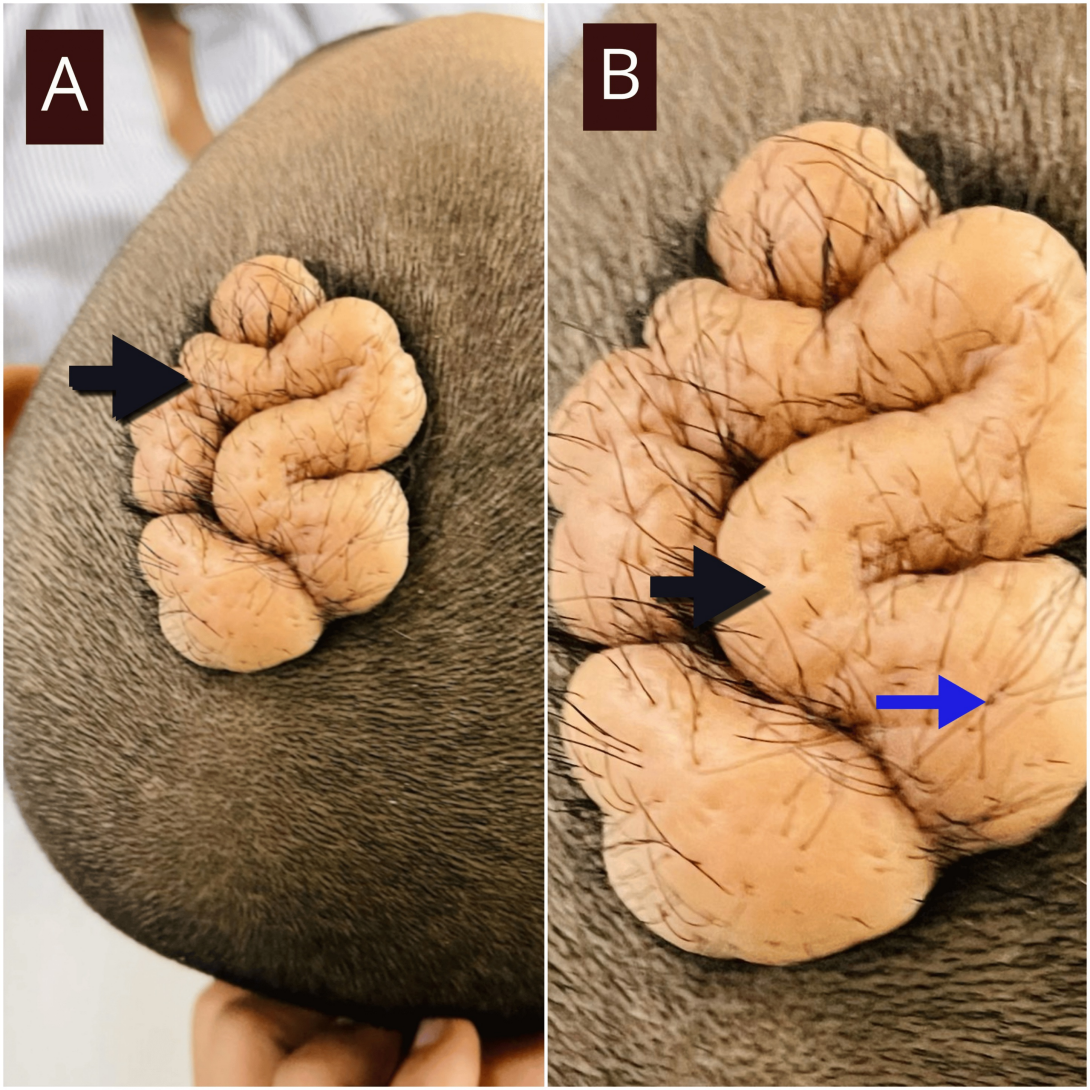
A, B: Sulcal-gyral surface pattern of the soft tissue mass on the left fronto-parietal region of the scalp (black arrows). Overlying hair growth can be seen (blue arrow)

An eight-year-old boy presented to the neurosurgery clinic with a painless, progressively enlarging scalp mass in the left fronto-parietal region. The lesion had been present since birth, with accelerated growth over the past two years. On examination, the mass measured 6×5×2 cm, was firm, mobile, non-tender, and not adherent to the calvarium (Figure 1). Overlying hair growth was present, and there was no warmth, erythema, or fluctuation. The child was otherwise well, and there was no family history of similar lesions.

**Figure 2:**
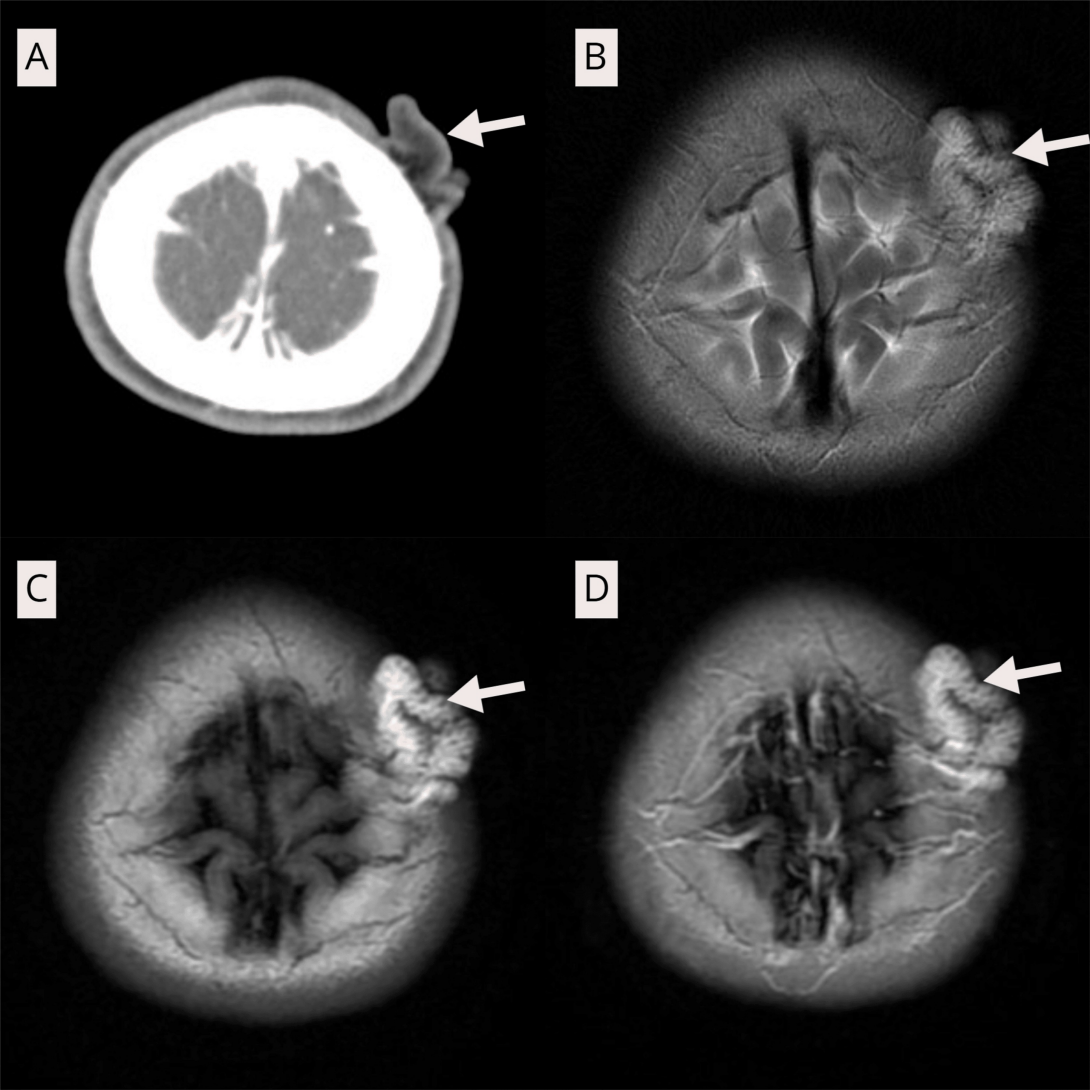
A: Post-contrast CT brain shows iso-hypodense extracranial mass without any vascular component (white arrow), B: T2-weighted image, C: T1-weighted image, D: T1 post-contrast images of MRI brain showing hyperintense and non-contrast enhancing lesion in the left fronto-parietal region (white arrows).

MRI demonstrated a subcutaneous, T1/T2-hyperintense lesion in the left fronto-parietal scalp without intracranial extension. Contrast-enhanced CT revealed a hypodense, non-enhancing growth beneath the skin. The surface exhibited a sulcal-gyral pattern corresponding to the clinical appearance (Figure 2). The preoperative radiologic impression was a benign subcutaneous fat-containing lesion; fibrous hamartoma of infancy was not included in the differential diagnosis. Differential considerations included scalp lipoma, lipoblastoma, lipofibromatosis, and lipofibromatous hamartoma.

**Figure 3:**
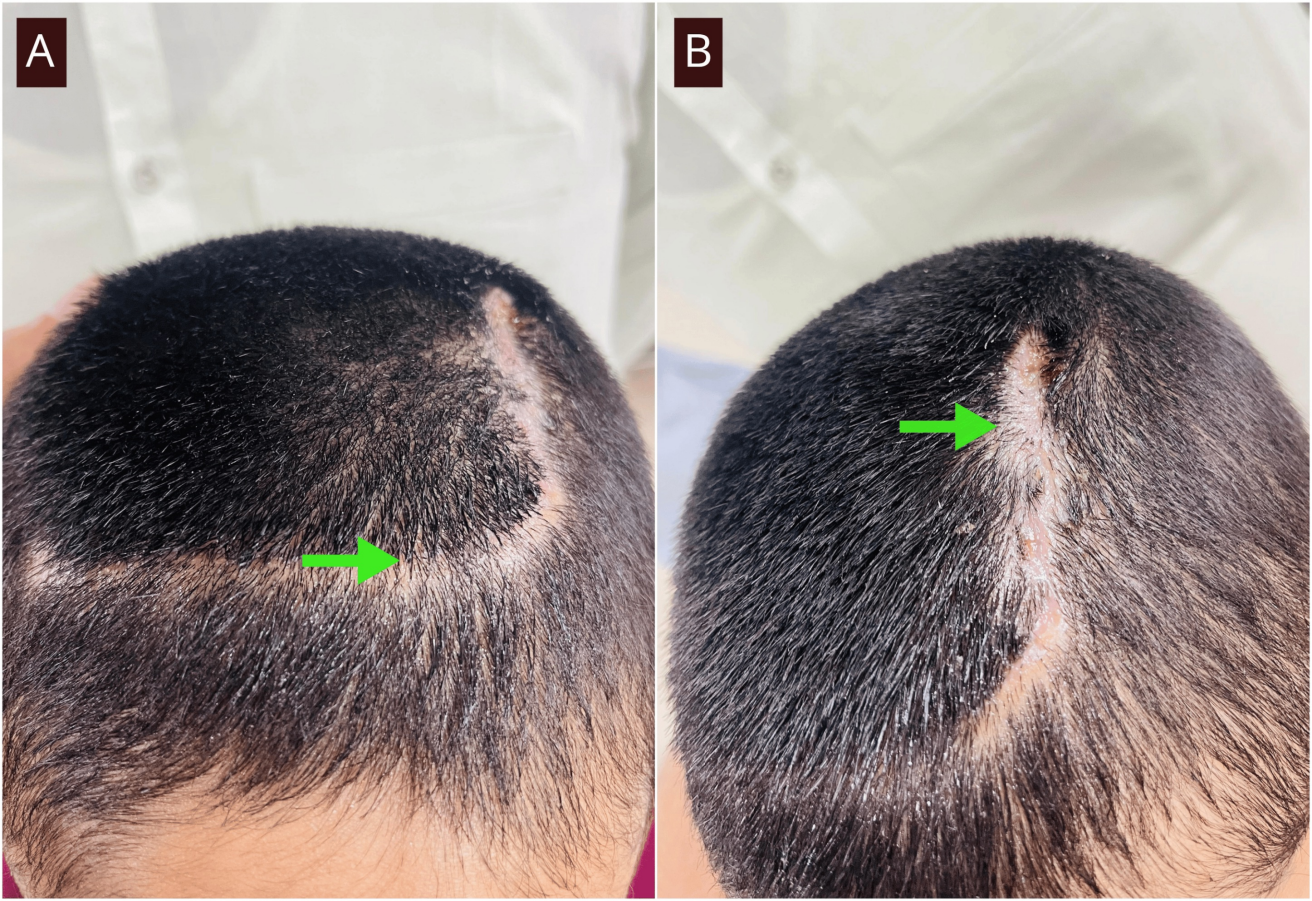
A, B: Postoperative images of scalp lesion with scar of rotational flap (green arrows).

After obtaining informed consent, complete excision was performed in collaboration with plastic surgery. The involved skin and thickened pericranium were excised with approximately 1 cm circumferential margins; no bony involvement was noted. The scalp defect was closed with a rotational flap and polypropylene 2-0 sutures. The post-operative course was uneventful, and sutures were removed on day 14 (Figure 3).

**Figure 4:**
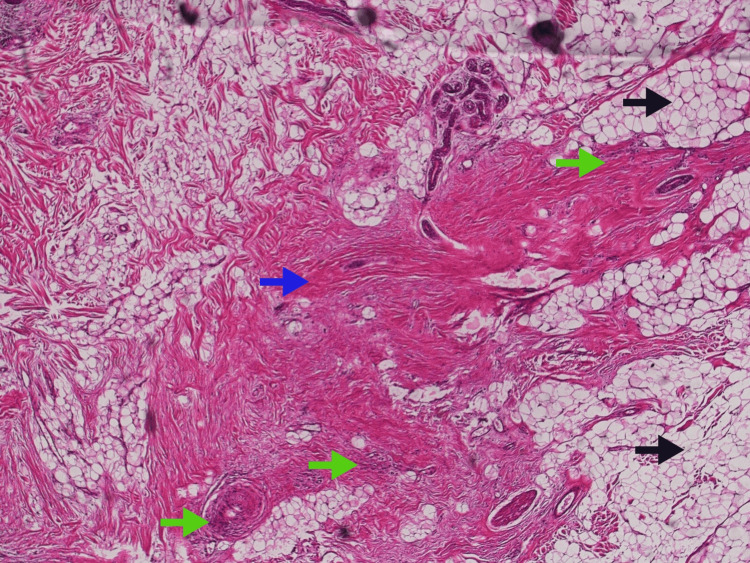
Hematoxylin and eosin (H&E) staining of the sections revealed a triphasic pattern of fibrous hamartoma of infancy (FHI) including mature adipose tissue (black arrow), fibrocollagenous tissue (blue arrow) and primitive spindle-to-stellate cells (green arrow).

Histopathology showed variable amounts of mature adipose tissue intersecting with fibrocollagenous fascicles and primitive spindle-to-stellate cells in a myxoid background arranged in an organoid pattern features consistent with fibrous hamartoma of infancy. No mitoses or necrosis were identified, and no malignancy was seen (Figure 4).

## Discussion

FHI usually presents as a painless subcutaneous mass on the trunk or proximal extremities; head-and-neck involvement is uncommon and scalp lesions are rarely described [2]. Our patient highlights two less typical features: an older age at presentation and a sulcal-gyral surface configuration that can mimic cerebral convolutions and complicate clinical assessment.

Large series reinforce the usual demographics and anatomic distribution. In the 145-case review by Al-Ibraheemi et al., most lesions arose on the trunk, axilla, or limbs, with a male predominance and a mean age at diagnosis of 15 months [2]. However, presentations beyond infancy are documented. Farho et al. reported an 11-year-old with a congenital lesion at the left iliac crest [7], and Wang et al. described a giant lower-extremity FHI in a 13-month-old that caused functional limitation [8]. These observations, together with our case, underscore that age and lesion morphology can vary.

Unusual anatomic sites may simulate other pathologies. Baek et al. reported FHI of the middle ear encasing the malleus without bony destruction [4]. Radiologically, FHI often shows a lobulated subcutaneous mass with internal fat and fibrous components; reported features include fatty streaks, calcifications on CT and mixed signals with whorled patterns on MRI [3]. The recognition of these patterns can narrow the differential diagnosis and guide surgical planning, while definitive diagnosis rests on histopathology [2,3].

Complete excision is the treatment of choice and is typically curative. In a nine-patient series, Smith et al. observed only one recurrence after complete resection, supporting the low but non-zero risk of relapse [9]. Careful margin assessment and clinicopathologic correlation are therefore recommended. In our case, complete excision with appropriate soft-tissue management achieved an uncomplicated recovery.

From a regional perspective, FHI appears to be rarely reported in Pakistan, and additional well-documented cases can refine awareness and diagnostic confidence [10]. Our case adds data from South Asia and draws attention to a scalp presentation with a distinctive sulcal-surface pattern that may influence preoperative considerations.

## Conclusions

FHI should be considered in the differential diagnosis of pediatric scalp masses, even beyond infancy. The recognition of unusual features, such as a sulcal-gyral surface pattern on imaging, may prevent misdiagnosis. The combination of a lobulated subcutaneous lesion with internal fat on imaging and triphasic histology supports accurate recognition. Complete excision is usually curative, with low recurrence rates when margins are adequate.
